# Comparison of Different Copromicroscopic Techniques in the Diagnosis of Intestinal and Respiratory Parasites of Naturally Infected Dogs and Cats

**DOI:** 10.3390/ani12192584

**Published:** 2022-09-27

**Authors:** Mariasole Colombo, Simone Morelli, Donatella Damiani, Maria Antonella Del Negro, Piermarino Milillo, Giulia Simonato, Alessandra Barlaam, Angela Di Cesare

**Affiliations:** 1 Faculty of Veterinary Medicine, University of Teramo, 64100 Teramo, Italy; 2 Azienda Sanitaria Locale (A.S.L.), 75100 Matera, Italy; 3 Department of Animal Medicine, Production and Health, University of Padua, 35020 Legnaro, Italy; 4 Department of Agriculture, Food, Natural Resources and Engineering (DAFNE), University of Foggia, 71121 Foggia, Italy

**Keywords:** copromicroscopic techniques, intestinal helminths, intestinal protozoa, cardio-pulmonary nematodes, flotation, Baermann, McMaster method, Mini-FLOTAC

## Abstract

**Simple Summary:**

Appropriate fecal examinations are very important for diagnosing parasitic diseases in dogs and cats. In this study, four different conventional copromicroscopic techniques (flotation, McMaster, Mini-FLOTAC, and Baermann) were evaluated for their performance in detecting intestinal and extra-intestinal parasitic elements in canine and feline feces. Stool samples from 100 dogs and 105 cats, respectively, were tested with the above techniques. Flotation and Mini-FLOTAC gave the best results in detecting intestinal and respiratory parasitoses by *Toxocara* spp., *Toxascaris leonina*, Ancylostomatidae, *Cystoisospora* spp., *Trichuris vulpis* and *Capillaria* spp., whereas the Baermann test was the best method for the detection of infections caused by metastrongyloids. These data provide useful information for veterinary clinicians on the most appropriate techniques to use during diagnostic paths in clinical settings.

**Abstract:**

Several copromicroscopic techniques, including tools belonging to the FLOTAC group, are available for the qualitative and/or quantitative diagnosis of canine and feline parasitoses. The present study was carried out to compare the diagnostic performance of different copromicroscopic methods for detecting common intestinal and extra-intestinal parasites of dogs and cats. Fecal samples of 100 dogs and 105 cats were randomly selected from different regions of Italy. All samples were subjected to conventional flotation, McMaster, Mini-FLOTAC, and Baermann. Fifty-six dogs and twenty-five cats were found positive to at least one technique, and, among them, flotation (55% and 20.9% of the dogs and cats, respectively) and Mini-FLOTAC (52% and 20.9% of the dogs and cats, respectively) detected the highest number of positive samples. Larvae of the feline metastrongyloids *Aelurostrongylus abstrusus* and *Troglostrongylus brevior* were identified only using the Baermann test in two (1.9%) and one (0.9%) cat respectively. No larvae were found with the Baermann examination of dog feces or any of the other methods. The present results show that the Mini-FLOTAC represents a possible alternative to conventional flotation in clinical settings for the detection of intestinal and respiratory parasites e.g., *Toxocara* spp., *Toxascaris leonina*, Ancylostomatidae, *Cystoisospora* spp., *Trichuris vulpis* and *Capillaria* spp., although Baermann’s test remains the most recommended technique for the diagnosis of infections caused by metastrongyloid lungworms.

## 1. Introduction

Dogs and cats can be infected by several intestinal and extra-intestinal parasites, which cause disease pictures of varying clinical severity and, in some cases, pose a significant public health risk. Copromicroscopy allow to diagnose infections caused by intestinal helminths (e.g., *Toxocara* spp., Ancylostomatidae, *Trichuris* spp., tapeworms), protozoa (e.g., *Cystoisospora* spp., *Giardia duodenalis*) [1], or cardio-respiratory nematodes, e.g., *Angiostrongylus vasorum*, *Aelurostrongylus abstrusus*, *Troglostrongylus brevior*, and *Capillaria* spp. [2,3,4].

Traditional qualitative tests, i.e., fecal flotation and Baermann’s method are simple and low-cost procedures used routinely to obtain a diagnosis in clinical settings.

The Modified McMaster technique allows a quantitative diagnosis of parasitic diseases, but it is scarcely used in daily small animal clinical practice due to its scarce sensitivity, especially in low-burden infections [1,5].

Alternative devices that belong to a group called FLOTAC have been more recently developed. FLOTAC and Mini-FLOTAC are both used in the quali-quantitative diagnosis of parasitic diseases caused by helminths and protozoa in dogs and cats. The FLOTAC methods require trained personnel and a laboratory equipped with a large volume centrifuge with a rotor for microtiter plate, unavailable in the majority of veterinary practices [6]. The Mini-FLOTAC is instead intended for rapid laboratory use and does not require centrifugation, being easy to use and suitable for laboratories with limited resources [1]. Both these devices are used in combination with the Fill-FLOTAC, a kit that allows ease of collection, processing of fecal samples, and the filling of the Mini-FLOTAC chambers [1,6]. Numerous studies have been conducted to evaluate the sensitivity and specificity of these diagnostic tools and data have shown that they can successfully detect intestinal parasites such as *Toxocara canis*, *Trichuris vulpis* and Ancylostomatidae [7,8]. Other studies have indicated that respiratory nematodes, including *Crenosoma vulpis*, *A. abstrusus,* and *T. brevior* larvae, may also be diagnosed using these devices [9,10,11,12].

The aim of the present study was to compare the diagnostic performance of the above different copromicroscopic techniques in naturally infected dogs and cats. In particular, the performance of mini-FLOTAC was compared to those of the traditional and gold standard techniques for the diagnosis of intestinal and extra-intestinal parasites, i.e., flotation [13,14] and the Baermann test [2,3,4].

## 2. Materials and Methods

### 2.1. Study Animals and Samples

Individual stool samples of 100 and 105 dogs and cats living in different regions of Italy were collected in the framework of their routine medical checks.

Samples were identified, numbered, and examined immediately after collection or shipped at refrigeration temperature and examined within 24 h post collection at the Laboratory of Parasitology and Parasitic Diseases of the Faculty of Veterinary Medicine of the University of Teramo. Each sample was subjected to flotation, McMaster technique, and Mini-FLOTAC using standard parameters (feces weight and NaCl solution specific gravity-S.G.-1.20). The Baermann test was performed using 2 g of feces.

### 2.2. Copromicroscopic Examinations

Flotation was performed by mixing 2 g of feces with 20 mL of saturated NaCl solution, until obtaining a homogeneous fecal suspension. The mixture was then filtered through a double layer of cotton gauze (250 µm mesh) and collected in a beaker. The fecal liquid was then transferred into a 15 mL Falcon tube with a conical bottom and centrifuged at 600× *g* for 5 min. After centrifugation, the same floating solution was added to the top of the tube to form a meniscus on which a coverslip was placed. After 3–5 min, the coverslip was placed on a slide and observed using a microscope at low-medium magnification (10×–20×) [15]. The parasitic elements detected were morphologically and morphometrically identified [16].

The McMaster examination was performed by mixing 2 g of feces in 28 mL of NaCl saturated solution until complete homogenization. The mixture was then filtered through a double layer of cotton gauze and collected in a beaker. Part of the filtered solution was taken using a Pasteur pipette and transferred to the McMaster chamber until its complete filling. After 5 min of flotation, the microscopic examination was carried out at 10× and 20× magnification [17]. The parasitic elements detected were identified using morphological and morphometrical keys. The number of eggs/oocysts counted in both grids was multiplied by 50 [16].

The fecal samples were also analyzed using the Mini-FLOTAC apparatus in combination with Fill-FLOTAC. Eighteen ml of NaCl solution were added to the graduated container of the Fill-FLOTAC kit and 2 g of feces were placed in the collector cone using a spatula (dilution 1:10). The fecal sample was homogenized by pushing the collector up and down and turning it to the left and right ten times. After turning over the Fill-FLOTAC 5 times to mix the suspension, the Mini-FLOTAC chambers were filled with the fecal suspension. After 10 min, the reading disc was rotated 90° clockwise to allow the separation of floating parasitic elements from fecal debris and observed using a microscope [1]. Parasite elements were counted in all 12 sections of the two chambers and the resulting number was multiplied by 5. The multiplication factor is obtained by dividing the dilution ratio by the volume of the two chambers (10:2 = 5) [1]. The detected parasitic elements were identified according to available identification keys [16].

The Baermann test was performed using 2 g of feces wrapped in a double layer of gauze and placed in a funnel filled with water at environmental temperature. After 12–24 h the fluid at the bottom of the funnel was collected in a falcon tube. The tube was then centrifuged at 600× *g* for 5 min, the supernatant was discarded, and the sediment was analyzed with a microscope [3]. The identification was done according to specific morphometric and morphological characteristics available in the literature [3].

### 2.3. Statistical Analysis

Sensitivity has been calculated for each technique, for each parasite with the following formula:

n. of positive for each technique/total number of animals positive to each parasite regardless of the technique × 100

The overall positivity percentage was calculated as follows: n. of animals positive to a given pathogen regardless of the method/ n. of animals examined × 100.

The negative predictive value (NPV) has been calculated using the following formula:

true negative/(false negative + true negative)

The total number of positive samples detected regardless of the methods was considered the diagnostic “gold” standard for each parasite species. Kappa (k) statistic was employed to determine to strength of agreement as follows: ≤0  =  poor, 0.01–0.20  =  slight, 0.21–0.40  =  fair, 0.41–0.60  =  moderate, 0.61–0.80  =  substantial, and 0.81–1  =  almost perfect.

## 3. Results

Overall, 56/100 dogs (56%) and 25/105 cats (23.8%) tested positive using at least one of the four diagnostic techniques.

### 3.1. Dogs

Of the 56 canine fecal samples positive for at least one parasite (56%), 55 (55%) scored positive when using flotation, 39 (39%) using McMaster, and 52 (52%) using Mini-FLOTAC, while none was positive when using the Baermann examination. Thirty-eight samples (67.9%) were positive using all the diagnostic techniques, i.e., 13 (23.2%) using flotation and Mini-FLOTAC, 1 (1.8%) using flotation and McMaster, 3 (5.4%) only using flotation, and 1 (1.8%) only using Mini-FLOTAC (Table 1 and Table 2).

Out of the 53 samples that scored positive using McMaster and/or Mini-FLOTAC, 38 (71.6%) were positive when using both techniques. Quantitative analyses results are shown in Table 3. Among positive dogs, 33 (58.9%) and 23 had a single or a mixed infection, respectively (Table 4 and Table 5).

### 3.2. Cats

Of the 105 examined cats, 22 (20.9%) scored positive when using flotation, 13 (12.3%) using McMaster, 22 (20.9%) using Mini-FLOTAC, and 4 (3.8%) using the Baermann test.

In total, 25 samples were found positive and, among them, 3 (12%) were positive using all the diagnostic techniques, 11 (44%) using flotation, McMaster, and Mini-FLOTAC, 12 (48%) using flotation and McMaster, 18 (72%) using flotation and Mini-FLOTAC, 13 (52%) using McMaster and Mini-FLOTAC, 3 (12%) only using flotation, 3 (8%) only using Mini-FLOTAC, and 3 only using the Baermann test (12%). Larvae of *A. abstrusus* and *T. brevior* were detected only using the Baermann test (Table 6 and Table 7).

Out of the 21 samples that scored positive when using McMaster and/or Mini-FLOTAC, 13 (61.9%) were positive using both techniques. Quantitative analyses results are shown in Table 8. Among positive cats, 21/25 (84%) had a single infection, while 4/25 (16%) had mixed infections (Table 9 and Table 10).

### 3.3. Statistical Analysis

Sensitivity and NPV values for each parasite and each technique are shown in Table 2 and Table 7 for dogs and cats respectively.

Kappa (k) statistic revealed an almost perfect K Cohen strength of agreement between flotation and mini-FLOTAC for all the parasites retrieved in this study in both cats and dogs. No statistical comparisons were possible between the Baermann method and other techniques (Table 11 and Table 12).

## 4. Discussion

The results of the present study confirm that Mini-FLOTAC is a sensitive and low-cost technique for the copromicroscopic diagnosis of canine and feline parasitoses [8,12] and that it may be considered an alternative to conventional flotation for most intestinal parasitoses of companion animals. At the same time, the Baermann test still remains the most recommended technique for detecting lungworm larvae.

The higher sensitivity of Mini-FLOTAC compared with the McMaster method for all the detected parasites is in line with the known sensitivity of the former method, i.e., 5 eggs per gram of feces (epg)/larvae per gram of feces (lpg)/oocysts per gram of feces (opg)/cysts per gram of feces (cpg) [1], compared to the ranges between 50 and 100 epg/lpg/opg/cpg known for the latter [6]. In agreement with the present results, previous studies have already shown that the Mini-FLOTAC has higher sensitivity and accuracy than the McMaster in the diagnosis of parasitic diseases in dogs and cats as well as other animals (e.g., goats, equines) [1,7,18,19,20].

Previous reports have indicated that the Mini-FLOTAC has absolutely higher diagnostic performance than classic copromicroscopy [8,10,12] and in the present study flotation and Mini-FLOTAC gave similar results. Nonetheless, these methods showed different sensitivity depending on the parasite elements in the stool samples. In dogs, the Mini-FLOTAC allowed the detection of a higher number of samples positive for *T. vulpis* compared to flotation, and it was the most sensitive technique also for the detection of other trichuroid eggs, i.e., *C. boehmi* in dogs and *C. aerophila* in cats. These findings are in line with previous results, i.e., higher sensitivity of Mini-FLOTAC for detecting *T. vulpis* eggs compared with the flotation in the tube, and for detecting *Capillaria plica* eggs upon sedimentation [7,21].

Flotation detected more positive samples of coccidian *Cystoisospora* spp. in both dogs and cats compared to Mini-FLOTAC. The NaCl solution, used in this study, is the most commonly recommended by Mini-FLOTAC producers for the detection of coccidian [1], and therefore these comparative results can be considered reliable. It should be taken into account that, to the best of the authors’ knowledge, these are the first comparative results between conventional flotation and Mini-FLOTAC available for intestinal coccidia of dogs and cats. Thus, further studies are needed to evaluate and compare the sensitivity of these methods for diagnosing coccidiosis in small animals. Flotation also detected more dogs and cats positive for Ancylostomatidae than Mini-FLOTAC. The NaCl solution is, again, the most suitable solution for the detection of hookworms with Mini-FLOTAC, as described previously [1], thus flotation is most probably the best option for these parasites. These results are in contrast with data obtained in a previous study, where better results were achieved in the detection of Ancylostomatidae using the Mini-FLOTAC. However, in the latter study Mini-FLOTAC was compared with flotation performed in a tube, that most probably is less sensitive than conventional flotation used here [7].

The detection of a taeniid egg in a cat fecal sample only using Mini-FLOTAC, and not with other techniques, was expected. Indeed, Mini-FLOTAC is currently indicated for the diagnosis of cestodes in dogs and cats, as flotation has some limits in terms of sensitivity in the detection of cestode eggs. Accordingly, in a recent study, the Mini-FLOTAC was more sensitive than other conventional copromicroscopic techniques in detecting cestode eggs in fox feces [22]. Thus, future studies are warranted to confirm its applicability in pet clinical practice.

In both dogs and cats, the Mini-FLOTAC showed the highest performance at identifying all the species involved in mixed infections.

The Baermann test is the gold standard for the diagnosis of cardiopulmonary parasitic nematodes due to the positive hydro-/thermo-tropism showed by their L1 when they are alive [3,4,23,24]. In the present study, the Baermann technique was the only method that allowed the detection of *A. abstrusus* and *T. brevior* larvae. This result is in contrast to the data of a recent study [12] where a higher performance of Mini-FLOTAC compared to the Baermann test was described, in terms of quantitative results. These contrasting results may be explained by the fact that the flotation solution herein used (i.e., NaCl solution S.G. 1.20) is not the most recommended for the detection of lungworm larvae with Mini-FLOTAC, which allows obtaining better results when used with zinc sulfate solution (S.G. 1.20). This might have influenced the performance of the Mini-FLOTAC technique and, therefore, has affected the recovery of parasitic elements [1]. Some salty solutions may indeed dehydrate the larvae, which may die without being able to migrate from the fecal matter, or they migrate, shrink, deform, and become difficult to identify [3]. In the present study, the Mini-FLOTAC has been performed using the NaCl solution because is the most commonly used in veterinary clinical practice, given the ease of finding, preparation, and low cost. Therefore, the choice of the solutions to be used in the Mini-FLOTAC must be accurate to minimize the possibility of false negative results. Accordingly, it has been reported that a more diluted flotation solution would reduce the chances of dehydration/rupture of the L1 using the Mini-FLOTAC, allowing a successful identification of parasitic elements [3,4,12]. Nevertheless, the Baermann test remains the gold standard for the detection of lungworms larvae in stool samples because: (i) it can be conducted in various settings (e.g., in veterinary practices and laboratories with basic equipment) [3]; (ii) it is cheaper than Mini-FLOTAC; (iii) its diagnostic performance does not depend on the choice of one solution rather than another, reducing the possibility of using an inappropriate solution in clinical settings; (iv) larvae eventually present in feces do not undergo any type of morphologic alteration and are easily found alive and viable in the sediment [3,4,23,24]. Moreover, in general, the lower sensitivity of the Mini-FLOTAC in the detection of the larvae could be attributed to the fact that it allows analyzing only 2 g of feces and, in cases where the parasitic load is very low, this amount may not be enough to provide reliable results. Although in the present study the same amount of feces was used for the Mini-FLOTAC and the Baermann technique to make a fair comparison, it should be considered that the Baermann is commonly performed with 5 or 10 g of feces, factually increasing the chances of detecting larvae even when only a few parasitic elements are present [3,4]. Therefore, the absence of larvae during Mini-FLOTAC examination does not allow excluding an infection with cardio-respiratory nematodes in dogs and cats, which must be confirmed with the Baermann test.

## 5. Conclusions

In conclusion, the present study confirmed the high performance of both flotation and mini-FLOTAC for the diagnosis of intestinal parasitoses in dogs and cats. Thus, the use of mini-FLOTAC may represent an alternative in clinical settings. In fact, despite this device being more expensive compared to flotation, it is an easy-to-use tool that can detect with relatively high sensitivity some intestinal (e.g., *Toxocara* spp. *Trichuris vulpis*, *Cystoisospora* spp.) and respiratory (*Capillaria* spp.) parasites. Further, large-scale surveys are needed to evaluate the performance of Mini-FLOTAC compared to the Baermann test. Nowadays the use of the Baermann test is still recommended as the most sensitive technique for the detection of lungworm infections in dogs and cats. In fact, a negative result obtained with the Mini-FLOTAC does not allow excluding an infection by *A. abstrusus* and/or *T. brevior*, and the Baermann test should be always performed when lungworm infections are suspected.

## Figures and Tables

**Table 1:** Number (n) and percentage (%) of dog fecal samples that scored positive using each technique out of the total number of tested samples (n tot).

Species	Flotation n/n Tot (%)	McMaster n/n Tot (%)	Mini-FLOTAC n/n Tot (%)
*Toxocara canis*	8/100 (8)	5/100 (5)	8/100 (8)
*Toxascaris leonina*	2/100 (2)	2/100 (2)	2/100 (2)
Ancylostomatidae	26/100 (26)	17/100 (17)	25/100 (25)
*Trichuris vulpis*	23/100 (23)	20/100 (20)	24/100 (24)
*Cystoisospora* spp.	5/100 (5)	2/100 (2)	2/100 (2)
*Capillaria aerophila*	14/100 (14)	3/100 (3)	13/100 (13)
*Capillaria boehmi*	3/100 (3)	1/100 (1)	4/100 (4)
Total *	55/100 (55)	39/100 (39)	52/100 (52)

* Total number of positive dogs with single or mixed infections.

**Table 2:** Number (n) and percentage (%) of dog fecal samples that scored positive using each technique compared to the overall positivity to a given parasite (n tot), to obtain the sensitivity of each technique for each parasite.

Species	Overall Positivity *	Flotation		Mc Master		Mini-FLOTAC	
	n/n Tot (%)	n/n Tot * (% ^#^)	NPV	n/n Tot * (% ^#^)	NPV	n/n Tot * (% ^#^)	NPV
*Toxocara canis*	9/100 (9)	8/9 (88.8)	0.98	5/9 (55.5)	0.95	8/9 (88.8)	0.98
*Toxascaris leonina*	2/100 (2)	2/2 (100)	1	2/2 (100)	1	2/2 (100)	1
Ancylostomatidae	27/100 (27)	26/27 (96.2)	0.98	17/26 (65.3)	0.89	25/27 (92.5)	0.97
*Trichuris vulpis*	26/100 (26)	23/26 (88.4)	0.96	20/26 (76.9)	0.92	24/26 (92.3)	0.97
*Cystoisospora*	5/100 (5)	5/5 (100)	1	2/5 (40)	0.96	2/5 (40)	0.96
*Capillaria aerophila*	15/100 (15)	14/15 (93.3)	0.98	3/15 (20)	0.87	13/15 (86.6)	0.97
*Capillaria boehmi*	4/100 (4)	3/4 (75)	0.98	1/4 (25)	0.96	4/4 (100)	1

* Regardless of the copromicroscopic technique used; ^#^ sensitivity; NPV: Negative predictive value.

**Table 3:** Number (n) of canine positive samples detected using quantitative techniques and mean value of epg (eggs per gram of feces) or opg (oocysts per gram of feces) retrieved for each parasite.

Species	McMaster		Mini-FLOTAC	
	n. Positive	epg/opg Mean Value	n. Positive	epg/opg Mean Value
*Toxocara canis*	5	257	8	157
*Toxascaris leonina*	2	492	2	550
Ancylostomatidae	17	332	25	240
*Trichuris vulpis*	20	157	24	87
*Cystoisospora* spp.	2	100	2	280
*Capillaria aerophila*	3	250	13	51
*Capillaria boehmi*	1	100	4	37

**Table 4:** Number of canine samples in which all the parasites involved in mixed infections have been detected using flotation, McMaster, and Mini-FLOTAC, respectively.

Total Mixed Infections ^#^ n/n Tot (%)	Flotation n/n Tot (%)	McMaster n/n Tot (%)	Mini-FLOTAC n/n Tot (%)
23/100 (23)	17/23 (73.9)	6/23 (26) ^1^	18/23 (78.2) ^2^

**^#^** Regardless of the copromicroscopic technique used. ^1^ McMaster gave a false negative result for 3 samples with mixed infections. ^2^ Mini-FLOTAC gave a false negative result for 1 sample with mixed infection.

**Table 5:** Species detected by each technique in co-infected dogs.

Samples n.	Flotation	Mini-FLOTAC	McMaster
21	Anc. + *T.v*. + *C.a.*	Anc. + *T.v*. + *C.a.*	Anc. + *T.v*.
28	Anc. + *C.a.*	Anc. + *C.a.*	Anc.
37	*C.b.*	*T.v.* + *C.b.*	-
50	Anc. + *C.a.*	Anc. + *C.a.*	Anc. + *C.a.*
53	*C.a.* + *C.b.*	Anc. + *C.a.* + *C.b.*	*C.a.* + *C.b.*
55	*T.v.* + *C.a.*	*T.c.* + *T.v.* + *C.a.*	*T.v.* + *C.a.*
57	*T.l.* + Anc.	*T.l.* + Anc.	*T.l.*
58	*T.c.* + Anc. + *C.a.*	-	-
60	Anc. + *T.v.*	Anc. + *T.v.*	Anc. + *T.v.*
63	Anc. + *T.v.*	Anc. + *T.v.*	Anc. + *T.v.*
66	Anc. + *T.v.*	Anc. + *T.v.*	Anc. + *T.v.*
67	Anc. + *T.v.*	Anc. + *T.v.*	Anc. + *T.v.*
68	*T.c.* + Anc.	*T.c.* + Anc.	-
70	Anc. + *T.v*. + *C.b.*	Anc. + *T.v*. + *C.b.*	*T.v*.
72	Anc. + *T.v*.	Anc. + *T.v*. + *C.b.*	Anc. + *T.v*.
73	*T.c.* + *T.v.*	*T.c.* + *T.v.*	*T.c.*
74	*Tl.* + *C.a.*	*Tl.* + *C.a.*	*T.l.*
81	Anc. + *Cyst.*	Anc. + *T.v*.	Anc.
82	Anc. + *Cyst.*	Anc. + *T.v*.	Anc. + *T.v*.
87	Anc. + *T.v*.	Anc. + *T.v*.	Anc. + *T.v*.
92	Anc. + *T.v*. + *C.a.*	Anc. + *C.a.*	*T.v*.
94	*T.c.* + *T.v.*	*T.c.* + *T.v.*	*T.c.*
97	Anc. + *T.v*.	*T.v.*	*T.v.*

Anc.: Ancylostomatidae; *T.v.: Trichuris vulpis; T.c.: Toxocara canis; T.l.: Toxascaris leonina; C.a.: Capillaria aerophila; C.b.: Capillaria boehmi; Cyst.: Cystoisospora* spp.

**Table 6:** Number (n) and percentage (%) of cat samples that scored positive using each technique compared to the overall number of samples (n tot).

Species	Flotation n/n Tot (%)	Mc Master n/n Tot (%)	Mini-FLOTAC n/n Tot (%)	Baermann n/n Tot (%)
*Toxocara cati*	9/105 (8.5)	3/105 (2.8)	9/105 (8.5)	-
*Toxascaris leonina*	1/105 (0.9)	0/105 (−)	1/105 (0.9)	-
Ancylostomatidae	4/105 (3.8)	3/105 (2.8)	3/105 (2.8)	-
*Cystoisospora* spp.	9/105 (8.5)	7/105 (6.6)	8/105 (7.6)	-
Cestode eggs	0/105 (−)	0/105 (−)	1/105 (0.9)	-
*Capillaria aerophila*	0/105 (−)	0/105 (−)	1/105 (0.9)	-
*Strongyloides stercoralis*	1/105 (0.9)	0/105 (−)	1/105 (0.9)	1/105 (0.9)
*Aelurostrongylus abstrusus*	0/105 (−)	0/105 (−)	0/105 (−)	2/105 (1.9)
*Troglostrongylus brevior*	0/105 (−)	0/105 (−)	0/105 (−)	1/105 (0.9)
Total *	22/105 (20.9)	13/105 (12.3)	22/105 (20.1)	4/105 (3.8)

* Total number of positive cats with single or mixed infections.

**Table 7:** Number (n) and percentage (%) of cat samples that scored positive using each technique compared to the overall positivity to a given parasite (n tot), to obtain the sensitivity of each technique for each parasite.

Species	Overall Positivity *	Flotation		Mc Master		Mini-FLOTAC		Baermann	
	n/n Tot (%)	n/n Tot * (% ^#^)	NPV	n/n Tot * (%^#^)	NPV	n/n Tot * (% ^#^)	NPV	n/n Tot * (% ^#^)	NPV
*Toxocara cati*	10/105 (9.5)	9/10 (90)	0.98	3/10 (30)	0.93	9/10 (90)	0.98	-	-
*Toxascaris leonina*	1/105 (0.9)	1/1 (100)	1	0/1 (−)	0.99	1/1 (100)	1	-	-
Ancylostomatidae	4/105 (3.8)	4/4 (100)	1	3/4 (75)	0.99	3/4 (75)	0.99	-	-
*Cystoisospora*	9/105 (8.5)	9/9 (100)	1	7/9 (77.7)	0.97	8/9 (88.8)	0.98	-	-
Cestodes	1/105 (0.9)	0/1 (−)	0.99	0/1 (−)	0.99	1/1 (100)	1	-	-
*Capillaria aerophila*	1/105 (0.9)	0/1 (−)	0.99	0/1 (−)	0.99	1/1 (100)	1	-	-
*Strongyloides stercoralis*	1/105 (0.9)	1/1 (100)	1	0/1 (−)	0.99	1/1 (100)	1	1/1 (100)	1
*Aelurostrongylus abstrusus*	2/105 (1.9)	0/2 (−)	0.98	0/2 (−)	0.98	0/2 (−)	0.98	2/2 (100)	1
*Troglostrongylus brevior*	1/105 (0.9)	0/1 (−)	0.99	0/1 (−)	0.99	0/1 (−)	0.99	1/1 (100)	1

* Regardless of the copromicroscopic technique used; ^#^ sensitivity; NPV: Negative predictive value.

**Table 8:** Number (n) of feline positive samples detected using quantitative techniques and mean value of epg (eggs per gram of feces) or opg (oocysts per gram of feces) retrieved for each parasite.

	Mini-FLOTAC		McMaster	
Species	n. Positive	epg/opg Mean Value	n. Positive	epg/opg Mean Value
*Toxocara cati*	9	142	3	350
*Toxascaris leonina*	1	50	1	50
Ancylostomatidae	3	640	3	1166
*Cystoisospora* spp.	8	1188	7	7657
Cestodes	1	5	0	-
*Capillaria aerophila*	1	5	0	-

**Table 9:** Number of samples in which all the parasites involved in mixed infections have been detected.

Total Mixed Infections ^#^ n/n Tot (%)	Flotation n/n Tot (%)	McMaster n/n Tot (%)	Mini-FLOTAC n/n Tot (%)	Baermann n/n Tot (%)
4/105 (3.8)	1/4 (25)	0/4 (0)	2/4 (50)	0/4 (0)

**^#^** Regardless of the copromicroscopic technique used.

**Table 10:** Species detected by each technique in co-infected cats.

Samples n.	Flotation	Mini-FLOTAC	McMaster
21	Anc.	Anc. + *C.a.*	Anc.
29	Anc. + *S.s.*	Anc. + *S.s.*	Anc.
96 *	*T.c.*	*T.c.*	*T.c.*
104 *	*T.c. + T.l.*	*T.c. + T.l.*	*T.c. + T.l.*

Anc.: Ancylostomatidae; *T.c.: Toxocara cati; T.l.: Toxascaris leonina; C.a.: Capillaria aerophila; S.s.: Strongyloides stercoralis*; * The sample scored positive also for *Aelurostrongylus abstrusus* using the Baermann test.

**Table 11:** Cohen’s kappa coefficient was calculated to assess the agreement between techniques employed in the present study for canine fecal samples.

Species		K Cohen (% *)	
	Flotation/McMaster	Flotation/Mini-FLOTAC	Mini-FLOTAC/McMaster
*Toxocara canis*	0.75 (97)	87 (98)	0.75 (97)
*Toxascaris leonina*	1 (100)	1 (100)	1 (100)
Ancylostomatidae	0.73 (91)	0.91 (97)	0.76 (92)
*Trichuris vulpis*	0.91 (97)	0.85 (95)	0.76 (92)
*Cystoisospora*	0.55 (97)	0.55 (97)	1 (100)
*Capillaria aerophila*	0.31 (89)	0.87 (97)	0.34 (90)
*Capillaria boehmi*	0.49 (98%)	0.85 (99)	0.39 (97)

* Percentage of agreement between the two techniques.

**Table 12:** Cohen’s kappa coefficient calculated to assess the agreement between techniques employed in the present study for feline fecal samples.

Species		K Cohen (% *)	
	Flotation/McMaster	Flotation/Mini-FLOTAC	McMaster/Mini-FLOTAC
*Toxocara cati*	0.47 (94)	0.94 (99)	0.47 (94)
*Toxascaris leonina*	- **	1 (100)	1 (100)
Ancylostomatidae	0.85 (99)	0.85 (99)	1 (100)
*Cystoisospora*	0.86 (98)	0.93 (99)	0.92 (99)
Cestodes	- **	- **	- **
*Capillaria aerophila*	- **	- **	- **
	Flotation/Baermann	Baermann/Mini-FLOTAC	Baermann/McMaster
*Strongyloides stercoralis*	1 (100)	- **	- **
*Aelurostrongylus abstrusus*	- **	- **	- **
*Troglostrongylus brevior*	- **	- **	- **

* percentage of agreement between the two techniques; ** Not computed;

## Data Availability

All study data are presented in the article.
